# Combining metaphase cytogenetics with single nucleotide polymorphism arrays can improve the diagnostic yield and identify prognosis more precisely in myelodysplastic syndromes

**DOI:** 10.1080/07853890.2022.2125173

**Published:** 2022-09-23

**Authors:** Yao Qin, Hang Zhang, Lin Feng, Haichen Wei, Yuling Wu, Chaoran Jiang, Zhihong Xu, Huanling Zhu, Ting Liu

**Affiliations:** a Department of Hematology, Institute of Hematology, West China Hospital, Sichuan University, Chengdu, P. R. China; b Sichuan Hua Xi Kindstar Medical Diagnostic Centre, Chengdu, Sichuan, P. R. China

**Keywords:** Metaphase cytogenetics, single-nucleotide polymorphism arrays, diagnosis, prognosis, myelodysplastic syndromes

## Abstract

**Background:**

Myelodysplastic syndromes (MDS) encompass a group of heterogeneous haematopoietic stem cell malignancies characterised by ineffective haematopoiesis, cytological aberrations, and a propensity for progression to acute myeloid leukaemia. Diagnosis and disease prognostic stratification are much based on genomic abnormalities. The traditional metaphase cytogenetics analysis (MC) can detect about 40–60% aberrations. Single-nucleotide polymorphism arrays (SNP-A) karyotyping can detect copy number variations with a higher resolution and has a unique advantage in detection of copy number neutral loss of heterozygosity (CN-LOH). Combining these two methods may improve the diagnostic efficiency and accuracy for MDS.

**Methods:**

We retrospectively analysed the data of 110 MDS patients diagnosed from January 2012 to December 2019 to compare the detection yield of chromosomal abnormalities by MC with by SNP-A, and the relationship between chromosomal abnormalities and prognosis.

**Results:**

Our results showed that SNP-A improved the detection yield of chromosomal aberrations compared with MC (74.5 vs. 55.5%, *p* < .001). In addition, the positive yield could be further improved by combining MC with SNP-A to 77.3%, compared with MC alone. Univariate analysis showed that age >65 years, bone marrow blasts ≥5%, with acquired CN-LOH, new aberrations detected by SNP-A, TGA value > the median (81.435 Mb), higher risk by IPSS-R-MC, higher risk by IPSS-R-SNP-A all had poorer prognosis. More critically, multivariable analysis showed that age >65 years and higher risk by IPSS-R-SNP-A were independent predictors of inferior OS in MDS patients.

**Conclusion:**

The combination of MC and SNP-A based karyotyping can further improve the diagnostic yield and provide more precise prognostic stratification in MDS patients. However, SNP-A may not completely replace MC because of its inability to detect balanced translocation and to detect different clones. From a practical point of view, we recommend the concurrent use of SNP-A and MC in the initial karyotypic evaluation for MDS patients on diagnosis and prognosis stratification.

**KEY MESSAGES**
- SNP-A based karyotyping can further improve the MDS diagnostic yield and provide more precise prognostic stratification in MDS patients.
- Acquired CN-LOH is a characteristic chromosomal aberration of MDS, which should be integrated to the diagnostic project of MDS.
- The concurrent use of SNP-A and MC in the initial karyotypic evaluation for MDS patients can be recommended.

## Introduction

Myelodysplastic syndromes (MDS) encompass a group of heterogeneous haematopoietic stem cell malignancies characterized by ineffective haematopoiesis, cytological dysplasia, and a propensity for progression to acute myeloid leukaemia (AML). Diagnosis and disease stratification are based on multiple factors including morphology of peripheral blood and bone marrow cells, flow cytometry, cytogenetics, and next-generation sequencing myeloid mutation studies [1]. The clonality of MDS had been confirmed by X chromosome inactivation and cytogenetic discoveries of non-randomly acquired chromosomal abnormalities [2]. Some chromosomal lesions are associated with different clinical phenotypes and can significantly affect prognose [3].

Metaphase cytogenetics (MC) is widely used as a “gold standard” for karyotypic analysis in a variety of blood diseases. It provides a whole-genome overview of structural and copy number variations at an average resolution of 5 ∼ 10 Mb. MC can detect balanced chromosomal changes including translocations or inversions, and unbalanced chromosomal changes including duplications and deletions. However, the sensitivity of MC is relatively low and the resolution depends on the location of the lesion with regard to the banding pattern. In addition, it is dependent on the cell proliferation in culture to obtain metaphases, only 40–50% of MDS patients can be found genomic aberrations [4–6]. Notably, most of chromosomal changes identified in MDS are unbalanced aberrations, leading to gains or losses in specific chromosomes [7, 8], and the non-informative cytogenetics due to an apparently normal or failed karyotype might lead to an inadequate estimation of the prognostic risk. Single-nucleotide polymorphism arrays (SNP-A) based karyotyping now has been commonly used as haematologic malignancies cytogenetic research [9, 10]. In contrast to MC, hybridisation of tumour DNA to arrays containing SNP allele variant specific probes allows detect not only in the copy number variations/aberrations (CNVs/CNAs), but also in the copy number neutral loss of heterozygosity (CN-LOH) at a much higher resolution. Acquired CN-LOH is frequently associated with MDS [11, 12] and cannot be detectable by conventional MC. What’s more, chromothripsis, which is a complex result of copy number alternating variation (normal, gain or loss) of a chromosome or chromosomal fragment, can be found by SNP-A [13]. In addition, compared with MC, the advantage of SNP-A-based karyotyping is not depending upon the availability of live dividing cells. However, the whole-genome SNP-A-based analysis for MDS now is only used as an optional item in the clinical guideline when a standard cytogenetics cannot be obtained or karyotype is normal [14].

This study is aimed at developing a rational diagnostic algorithm based on combining MC with SNP-A technical advantages. We hypothesized that identification of new aberrations with the use of SNP-A may complement MC and improve the diagnostic yield and prognostic stratification of MDS.

## Materials and methods

### Patients

We retrospectively analysed the data of 110 newly diagnosed primary MDS patients hospitalised in the Department of Haematology, West China Hospital of Sichuan University during January 2012 to December 2019, and the median follow-up time was 23.0 months (2.0–96.0 months). The clinical characteristics of these patients were listed in Table 1, including 72 males and 38 females, mean age 58 (range, 16–84 years). According to World Health Organization (WHO) classification of myeloid neoplasms and acute leukaemia in 2016 [3], seven patients (6.4%) were diagnosed as myelodysplastic syndromes with single lineage dysplasia (MDS-SLD), 20 patients (18.2%) as myelodysplastic syndromes with multilineage dysplasia (MDS-MLD), 29 patients (26.4%) as myelodysplastic syndromes with excess blasts-1 (MDS-EB1), 28 patients (25.4%) as myelodysplastic syndromes with excess blasts-2 (MDS-EB2), four patients (3.6%) as myelodysplastic syndromes with isolated del(5q) (5q- syndrome), and 22 patients (20.0%) as myelodysplastic syndromes unclassifiable (MDS-U). According to Revised International Prognostic Scoring System (IPSS-R) evaluation [15], the prognostic stratification was scored as very low risk 1 patient (1.0%), low risk 14 patients (12,7%), intermediate risk 37 patients (33.6%), high risk 22 patients (20.0%), and very high risk 36 patients (32.7%). Patients received treatments that included supportive therapy, low intensity chemotherapy, high-intensity chemotherapy, and haematopoietic stem cell transplantation (Supplementary Table 1). As conventional diagnostic process, informed consent for sample collection was obtained from patients, and the study protocol was approved by the Ethics Committee of West China Hospital of Sichuan University（No. 2022-777). The clinical and laboratory data of all patients were collected from the Hospital Information System (HIS) and the Laboratory Information System (LIS). The patient’ survival conditions were followed up *via* hospital medical records, outpatient visits, and telephone calls.

**Table 1.** Baseline characteristics of 110 MDS patients.

Patient characteristics	Value
Age	
Median age, range (years)	58 (16–84)
Sex	
Male/female (%)	65.5/34.5
WHO classification (%)	
MDS-SLD	7 (6.4)
MDS-MLD	20 (18.2)
5q- syndrome	4 (3.6)
MDS-EB1	29 (26.4)
MDS-EB2	28 (25.4)
MDS-U	22 (20.0)
IPSS-R, *n* (%)	
Very low	1 (1.0)
Low	14 (12.7)
Intermediate	37 (33.6)
High	22 (20.0)
Very high	36 (32.7)
AML transformation, *n* (%)	
Yes	11 (10.0)
No	99 (90.0)

*Abbreviations*: MDS-SLD: myelodysplastic syndromes with single lineage dysplasia; MDS-MLD: myelodysplastic syndromes with multilineage dysplasia; 5q- syndrome: myelodysplastic syndromes with isolated del(5q); MDS-EB1: myelodysplastic syndromes with excess blasts-1; MDS-EB2: myelodysplastic syndromes with excess blasts-2; MDS-U: myelodysplastic syndromes: unclassifiable; IPSS-R: revised international prognostic scoring system; AML: acute myeloid leukaemia; n: number.

### Metaphase cytogenetic analysis

Bone marrow aspirates were subjected to cytogenetic analysis according to standard methods. Chromosome preparations were performed by trypsin and Giemsa (GTG) for G-banding, and karyotypes were described based on the International System for Human Cytogenetic Nomenclature (2013) [16]. At least 20 metaphases were analysed for each patient. Patients with no growth of the cell culture for MC are defined as noninformative cases.

### SNP-A analysis

DNA from bone marrow samples was extracted using QIAamp DNA Blood Mini Kit (QIAGEN, Suzhou, China). DNA quantity and purity were assessed by Nanodrop 2000 spectrophotometry (Thermo Scientific, USA). The quality of DNA was detected by gel electrophoresis. The high-density array assay (CytoScan 750 K arrays and reagents, Thermo Fishher Scientific) was run according to the manufacturer’s protocol. The average resolution of this microarray is 100 kb. Briefly, 250 ng of patient DNA was digested with Nsp1, amplified with TITANIUM Taq DNA polymerase (Clontech), fragmented with fragmentation reagent, and labelled with biotin end-labelled nucleotides. The DNA was hybridised to the microarray for 16 h, washed on the Gene Chip Fluidics Station 450, stained with Gene Chip Stain Reagents, and scanned on the Gene Chip Scanner 3000Dx v.2 (Thermo Scientific, USA). Total genomic aberrations (TGA) were calculated based on total length of DNA in Mb of somatic aberration (copy neutral loss of heterozygosity and copy number aberrations). Data analysis was performed using Chromosome Analysis Suite software (ChAS, Thermo Fishher Scientific) version 4.0. The identical thresholds of aberrations reported via SNP-A were according to manufacturer’s recommendation: loss≧400kb, gain≧400kb, and mosaic clone≧10%. CN-LOH can be classified by either its origin or its location. The distinction between acquired somatic CN-LOH with constitutional, nonclonal derived CN-LOH could be identified by comparison of the DNA from bone marrow with from nail or hair of the MDS patients. Only homozygosity regions were interpreted as acquired CN-LOH, if the regions were >5 Mb and located on the terminal end of the chromosome. All identified aberrations must be confirmed on the Atlas of Genetics and Cytogenetics in Oncology and Haematology (http://atlasgeneticsoncology.org/TAnomalies/Anomliste.html) and literature reviewed.

### Statistical analysis

SPSS version 25.0 was used for statistical analysis. Chi-square test and Fisher exact test and were used to compare categorical variables. Overall survival (OS) was measured from diagnosis to death from any cause. OS curves were compared by the log-rank test and plotted by the Kaplan–Meier method. multivariable analyses of OS were performed using Cox proportional hazards model. All *p* values were 2-sided, and *p* value <.05 was viewed as statistically significant. Chromosomal figures were drawn by R software version 3.6.1 and OS curves were drawn by GraphPad Prism software version 7.00.

## Results

### Metaphase cytogenetics-based karyotyping

**Figure 1.**
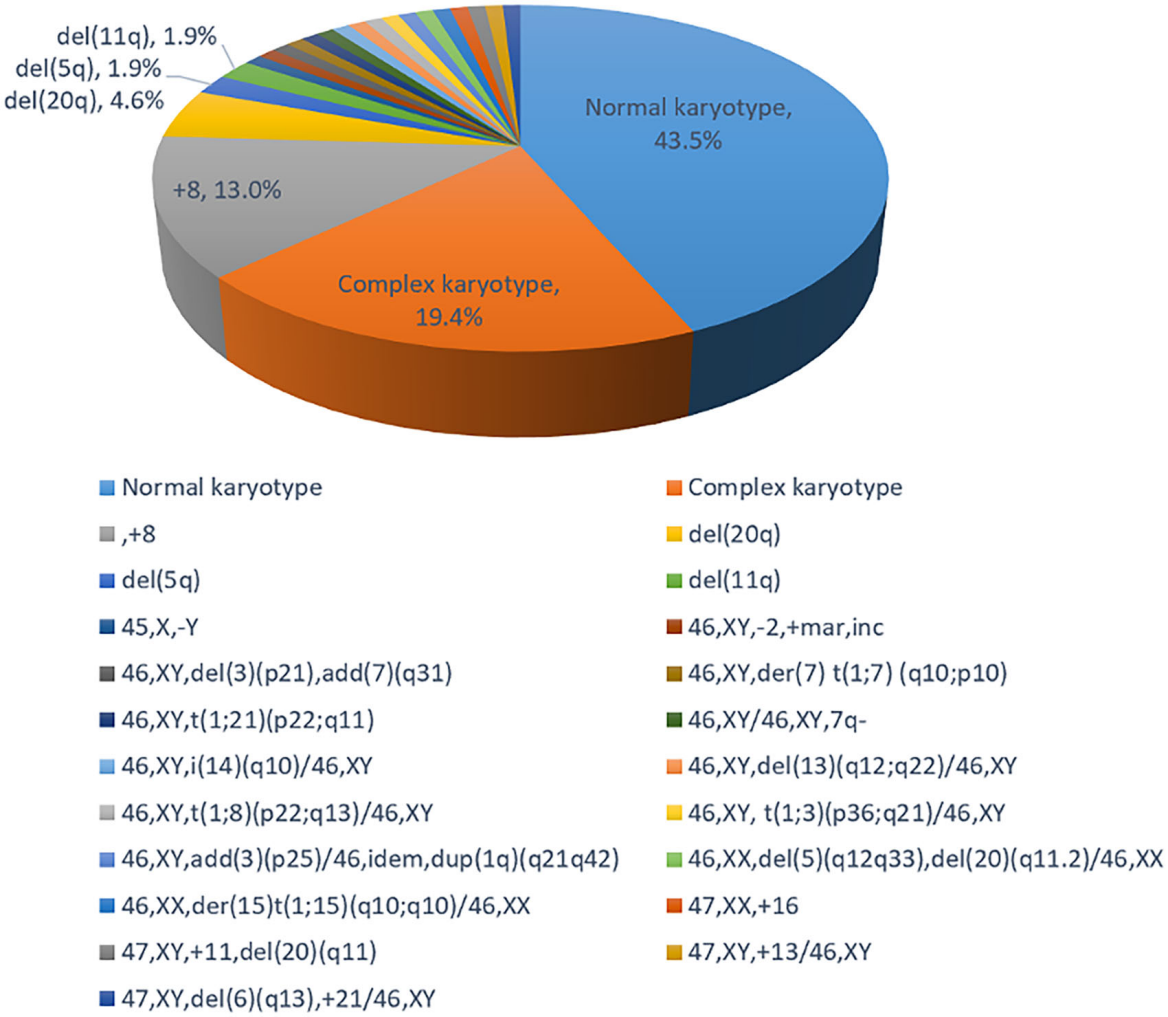
Cytogenetic characteristics of 108 patients with myelodysplastic syndromes.

Among the 110 MDS patients detected by MC, 47 cases (42.7%) were normal karyotype, 61 cases (55.5%) were abnormal karyotype, and two cases (1.8%) were noninformative. Among 61 patients with abnormal karyotype, there were two cases (1.8%) of simple balanced structural abnormality, eight cases (7.3%) of unbalanced structural abnormality, one case (0.9%) of hypodiploid, eight cases (7.3%) of hyperdiploid, 21 cases (19.1%) of complex karyotype, and 21 cases (19.1%) of chimeric karyotype (Supplementary Table 2). All 21 complex karyotype cases had four or more chromosomal abnormalities. Among 21 chimeric karyotypes, 19 (90.5%) were normal karyotype with abnormal karyotype, while two (9.5%) were abnormal karyotype with abnormal karyotype (Supplementary Table 3). All chromosomal abnormalities based on MC were shown in Figure 1.

### SNP-A-based karyotyping

**Figure 2.**
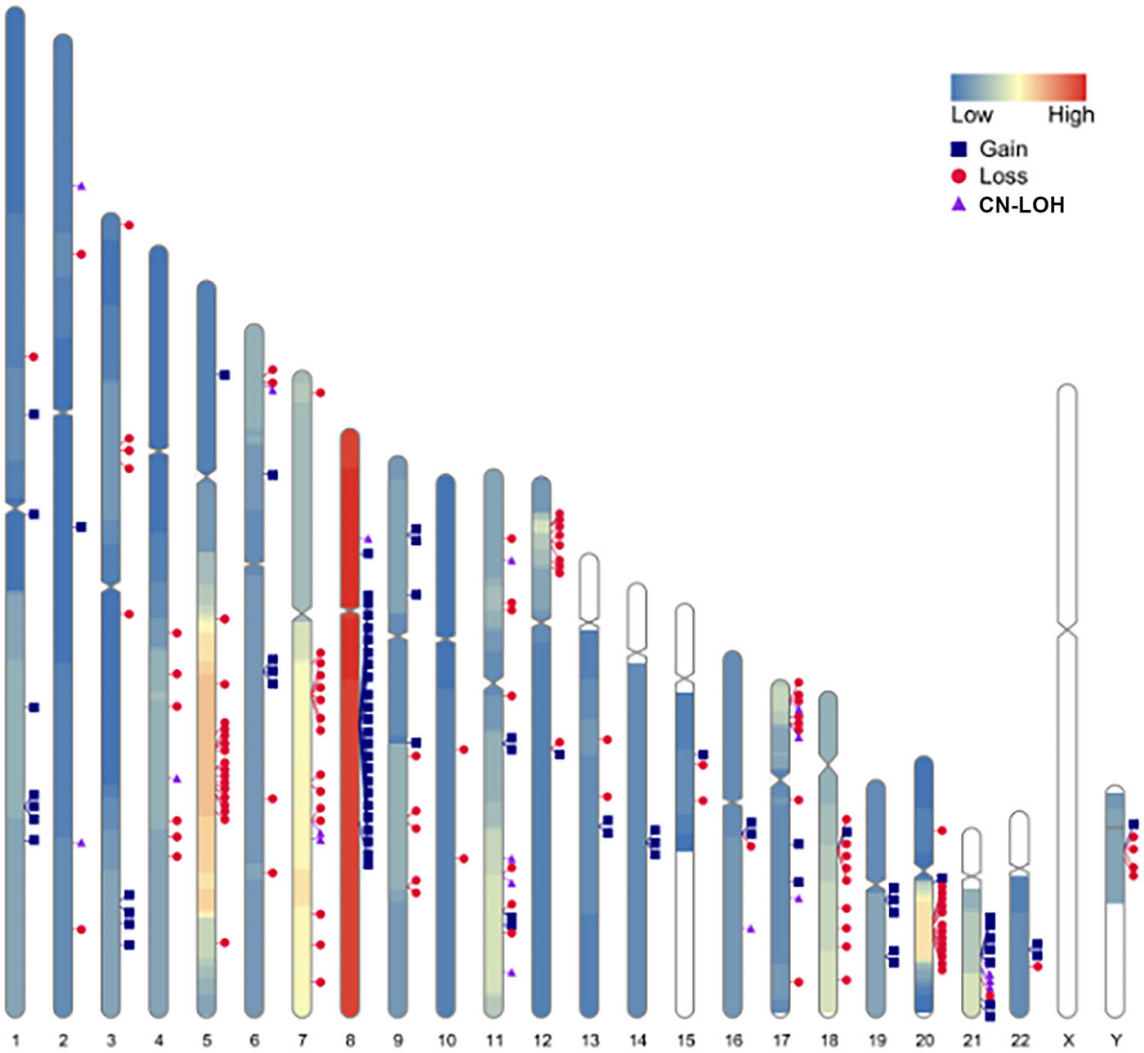
Frequency and location of gain, loss, and CN-LOH detected by SNP-A on each chromosome in 110 patients with myelodysplastic syndrome. Colourlessness on chromosomes indicates the absence of chromosomal aberrations in the region; Low indicates low frequency of chromosomal aberrations; high indicates high frequency of chromosomal aberrations; each marker indicates the intermediate site of chromosomal aberrations. CN-LOH: copy number neutral loss of heterozygosity.

All 110 MDS patients bone marrow samples were parallelly detected by SNP-A. Among them, 25 cases (25.5%) had no chromosomal abnormities and 82 cases (74.5%) showed chromosomal abnormities (Supplementary Table 4). There were 42 patients (38.2%) with gain/gainmosaic, most frequently found on chromosomes 8, 1, and 21 with 23, 7, and 6 cases, respectively. 53 (48.2%) patients with loss/lossmosaic were most frequently found on chromosomes 5, 7, and 20 with 21, 19, and 15 cases, respectively. There were 21 cases (19.1%) with acquired CN-LOH as either the sole abnormality or as a concurrent aberration, most of acquired CN-LOH were on chromosomes 17, 7, 11, and 21, with 5, 3, 3, and 3 cases, respectively (Figure 2). There were 25 patients (22.7%) with complex karyotype. 14 patients (12.7%) were identified chromothripsis, mostly on chromosomes 20, 3, 6, 9, and 21 with 5, 2, 2, 2 and 2 cases, respectively. All chromothripsis occurred in the MDS patients with complex karyotypes, included eight MDS-EB2, four MDS-EB1, and two MDS-MLD cases.

### Comparison of MC and SNP-A-based karyotyping

We compared the results of MC and SNP-A-based karyotyping in 110 MDS patients. Sixty-one cases (55.5%) had abnormal karyotypes by MC analysis and 82 cases (74.5%) had chromosomal abnormities by SNP-A analysis. SNP-A increased the detection yield of chromosomal aberrations (55.5 vs. 74.5%, *p* < .001). In addition, three patients were detected chromosomal abnormalities only by MC, these abnormalities were “46, XY [11]/46, XY, 7q-[1]”, “46, XY, −2, +mar, inc[4]”, and “46, XY, t (1; 8) (p22; q13)[7]/46, XY[13]”, respectively (Supplementary Table 5). We speculate that the possible reasons were not detectable by SNP-A were anomalies present in very small clones in the first two patients, and a balanced translocation may be in the third case. By combining MC with SNP-A, 85 cases (77.3%) of chromosomal abnormities were detected, the positive rate of chromosomal aberrations could be increased (55.5 vs. 77.3%, *p* < .001) (Table 2).

**Table 2.** Comparison of chromosomal abnormities between MC and MC combining with SNP-A in 110 MDS patients.

MC	*n* (%)	MC + SNP-A	*n* (%)	*p* Value
Noninformative	2 (1.8)	Normal	25(22.7)	
Normal	47(42.7)	Abnormal	24(21.8)	
Abnormal	61(55.5)	No additional	28(25.5)	
Abnormal		Additional	33(30.0)	
All abnormal	61(55.5)	All abnormal	85(77.3)	<.001

*Abbreviations*: MC: metaphase cytogenetics; SNP-A: single nucleotide polymorphism arrays; *n*: number.

The distribution of new chromosomal abnormities on each chromosome detected by SNP-A in patients is shown in Supplementary Table 6. By SNP-A, 24 of 49 (49.0%) patients with normal/noninformative MC karyotypes were detected new chromosomal abnormities; 33 of 61 (54.1%) patients with abnormal MC results were detected new chromosomal lesions; two patients with MC noninformative were detected chromosomal abnormities. Among them, a single new chromosomal abnormality was found in 33 patients detected by SNP-A, which was most common on chromosomes 7, 8, 11, and 20. Two new chromosomal abnormalities was identified by SNP-A in 6 cases. In addition, 3 or more new abnormalities were mainly found in 16 previous abnormal MC cases, 1 normal MC case, and 1 noninformative MC case. 21 of 110 patients (19.1%) were detected acquired CN-LOH by SNP-A. Among them, 8 of 47 patients (17.0%) were with normal MC results, involving chromosomes 11, 4, 6, 7, 8, 17 and 21, respectively. 11 of 61 patients (18.0%) were with abnormal MC results, including chromosomes 17, 11, 2, 7, 16, 19 and 21, respectively. For complex karyotype, 21 patients (19.1%) were detected by MC, and 25 patients (22.7%) were detected by SNP-A. The frequency and types of abnormalities detected by MC or SNP-A were showed in Supplemental Figure 1 and Supplementary Table 7. New aberrations by SNP-A are defined as MC undetectable loss, gain, and CN-LOH. Surprisingly, new chromosomal aberrations were found in up to 51.8% of patients.

### SNP-A on prognosis in MDS

**Figure 3.**
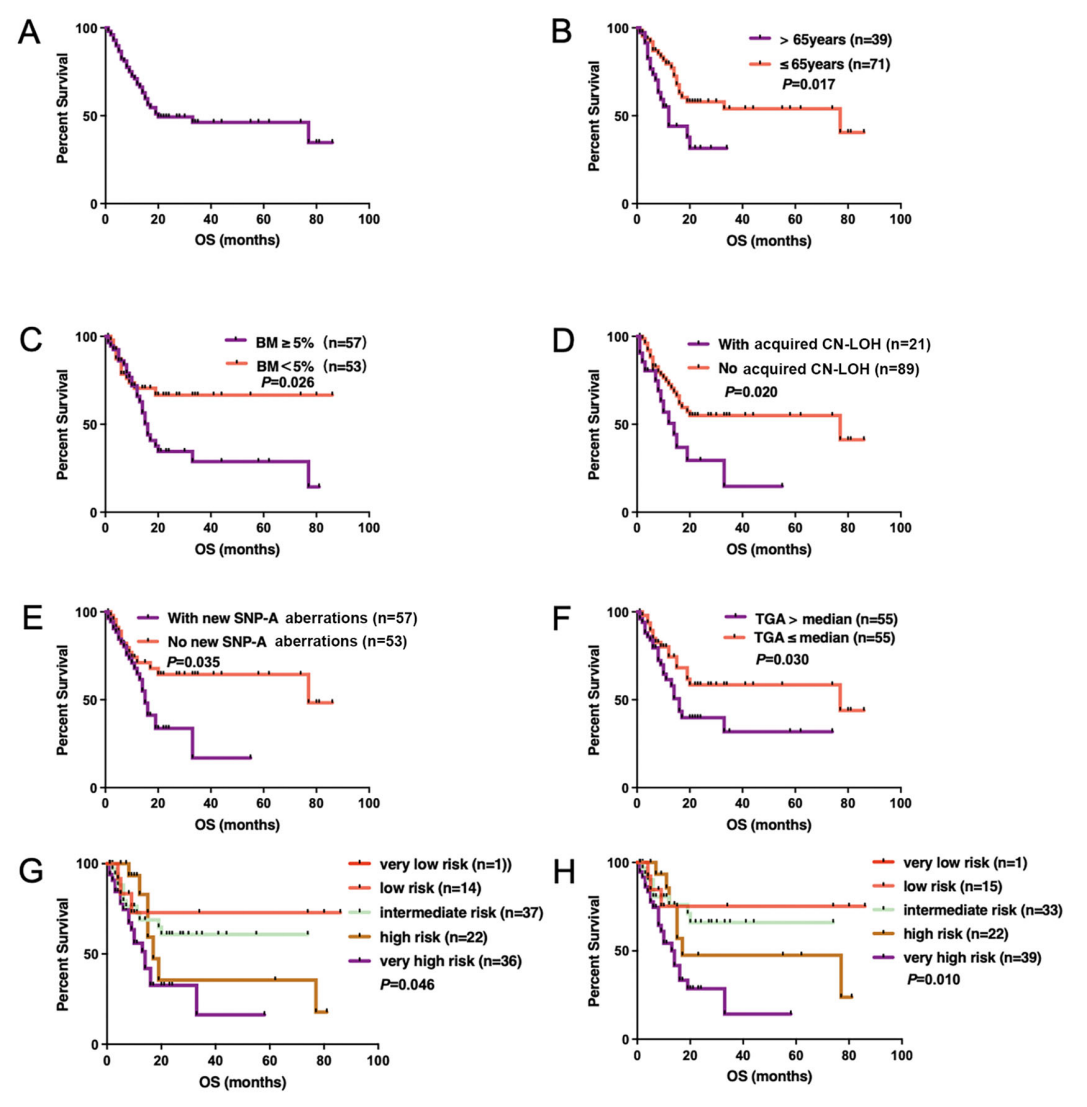
The overall survival (OS) curve of univariate analysis of all MDS patients. (A) OS curve of all MDS patients; (B) age; (C) bone marrow blasts; (D) acquired CN-LOH; (E) new SNP-A aberration; (F) TGA value; (G) survival by IPSS-R based on MC results; (H) survival by IPSS-R based on SNP-A results. MSD: myelodysplastic syndromes; SNP-A: single nucleotide polymorphism arrays; CN-LOH: copy number neutral loss of heterozygosity; TGA: total genomic aberrations; IPSS-R: Revised International Prognostic Scoring System; MC: metaphase cytogenetics.

Of significant importance for the clinical applicability of SNP-A is whether the increased cytogenetic yield can be translated to more precise prognosis stratification? In our study, 110 patients with MDS were followed up for 2.0–96.0 months (median 23.0 months). The median OS of all patients was 20.0 (95%CI 0–53.6) months, and the two-year OS rate was 48.9% (Figure 3(A)). Univariate analysis showed (Supplementary Table 8): the median OS of MDS patients aged >65 years vs. ≤65 years were 12.0 vs. 77.0 months (*p* = .017) (Figure 3(B)); the median OS of MDS patients with bone marrow blasts ≥5 vs. <5% were 15.0 months vs. not reached (*p* = .026) (Figure 3(C)); the median OS of patients with acquired CN-LOH detected by SNP-A was worse than that of patients who had no CN-LOH (14.0 vs. 77.0 months; *p* = .020) (Figure 3(D)); the median OS of patients with new aberrations detected by SNP-A was significantly inferior than that of patients without new aberrations detected (15.0 vs. 77.0 months; *p* = .035) (Figure 3(E)). In addition, we also estimated the median value of total genomic aberrations (TGA) which was 81.435 Mb in our study. Patients with TGA above 81.435 Mb (range, 0–2172.22) had inferior OS compared to those below the value (14.0 vs 77.0 months, *p* = .030) (Figure 3(F)). Finally, with IPSS-R prognostic stratification, both either based on MC results (IPSS-R-MC), or based on SNP-A results (IPSS-R-SNP-A) had significant effects, *p* = .046 and .010, respectively (Figure 3(G,H)). More importantly, multivariable analysis showed that aged >65 years and risk stratification by IPSS-R-SNP-A were independent predictors of inferior OS in MDS patients (Supplementary Table 9).

## Discussion

MDS patients are highly heterogeneous and present with varying clinical manifestations, prognostic stratification, and risks of AML transformation. Nowadays, the diagnosis of MDS is based on the WHO classification in 2016 [3] and the revised international prognostic scoring system(IPSS-R) in 2012 [15], which includes cytogenetic abnormalities, percentage of bone marrow blasts, and cytopenia with unilineage or multilineage blood cells. Cytogenetic findings have an established role in the diagnosis and assessment of prognosis of MDS and are emerging as an important factor in treatment selection and response monitoring. Conventional cytogenetic analysis techniques include MC and fluorescence *in situ* hybridization (FISH). The standard MC technique can only detect chromosomal rearrangements of more than 10 Mb in size. Furthermore, chromosome banding analysis is dependent on the cell proliferation of MDS clones in culture to obtain metaphases. Thus, MC technique will miss many important chromosome abnormalities, resulting in genomic aberrations detectable in only 40–50% of MDS patients [6]. FISH may complement the metaphase cytogenetics study; however, its utility is limited to the detection of specific lesions [17]. SNP-A has a higher analytical resolution than MC, it can detect submicroscopic or cryptic deletions or duplications, especially in patients with normal/noninformative MC karyotypes. Despite of the high resolution and comparable sensitivity of SNP-A, SNP-A-based karyotyping for MDS now is only recommended as an optional item in the clinical guideline when a standard cytogenetics cannot be obtained or karyotype is normal [14].

The clinical applicability of SNP-A analysis for MDS has been confirmed in some previous studies. Gondek et al. first applied Affymetrix 50 K SNP-A to 66 and 72 patients with MDS and showed that chromosomal abnormalities were present in 82% patients, of which, 68% were in normal MC karyotype and 81% were in abnormal MC karyotype. At the same time, they found that 33% MDS patients had acquired CN-LOH abnormalities [18, 19]. Subsequently, using 250 K SNP-A, Gondek et al. found chromosomal abnormalities in approximately 3/4 of MDS, myelodysplastic/myeloproliferative diseases (MDS/MPD) and secondary AML (sAML). In addition, segmental CN-LOH abnormities were found in 20% of MDS, 23% of sAML, and 35% of MDS/MPD patients [11]. A prospective study of self-matched pairs of bone marrow cells to buccal cells for 51 MDS patients by 250 K SNP-A found somatically acquired chromosomal aberrations in 41% patients with MDS [20]. Tiu el al. studied 250 MDS patients with self-matched pairs bone marrow cells to CD3+ lymphocytes by 250 K SNP-A. The results demonstrated that combining MC with SNP-A led to a higher diagnostic yield of chromosomal aberrations compared with MC alone (74 vs 44%). It also showed that the new chromosomal lesions detected by SNP-A predicted poor prognosis of MDS by univariate and multivariate analyses [7]. In a study from China for 162 MDS patients with 750 K SNP-A showed that 76 patients (46.9%) had chromosomal gains with +8 (17.9%) most common, 101 patients (62.3%) had chromosomal losses with 5q- (21.0%) most common, and 51 patients (31.5%) had acquired CN-LOH abnormalities with CN-LOH (7q) (4.94%) and CN-LOH(17p) (4.32%) most common [21]. Compared with MC karyotyping, our results showed that SNP-A could improve the detection yield of chromosomal aberrations (74.5 vs. 55.5%, *p* < .001), and the detection yield could be further improved by combining MC with SNP-A (77.3 vs. 55.5%, *p* < .001). Therefore, as a complement to MC, SNP-A high-resolution technique provides more accurate detection to cryptic chromosomal abnormities.

Acquired CN-LOHs have been described in several haematological disorders, including MDS, myeloproliferative diseases, and AML [7, 22–24]. A previous study found CN-LOH were frequently detected on chromosomes 6, 11, 4, and 7 [11]. Acquired CN-LOH is likely the result of mitotic recombination and appears to be a common event in MDS [20]. Besides, acquired CN-LOH helps to identify gene mutations associated with MDS and related diseases. Such as, CN-LOH (4q24) in MDS promoted the discovery of TET2 gene mutation [25]; CN-LOH (11q) in CMML promoted the identification of CBL gene mutation [26, 27], and CN-LOH (17p) in MDS/sAML promoted the confirmation of TP53 gene mutation [28]. In our study, 21 patients (19.1%) with acquired CN-LOH were detected by SNP-A, mostly on chromosomes 17, 7, 11 and 21, which were consistent with those found by Tiu et al. [7]. Therefore, CN-LOH abnormalities can make up for the inability of MC karyotyping.

In addition, chromothripsis is a unique type of genomic instability and plays a vital role in the development of cancer [29]. In haematopoietic neoplasms, chromothripsis was linked to poor prognosis and specific genetic alterations: complex karyotype, 5q deletions, and loss of TP53 [30]. Gao et al. identified chromothripsis in nine AML and two MDS cases, and noted that all chromothripsis-positive AML cases were with MDS-related changes. Chromothripsis in AML-MDS most frequently involves chromosomes eight and 11 with consequent amplification of either MYC or KMT2A [31]. Abáigar et al. found that three high-risk MDS patients displayed chromothripsis involving exclusively chromosome 13 and affecting some cancer genes: FLT3, BRCA2 and RB1, and all of them carried TP53 mutations [32]. In our study, chromothripsis were detected by SNP-A in 14 patients (12.7%) with mostly on chromosomes 20, 3, 6, 9, and 21. All chromothripsis occurred in the MDS patients with complex karyotypes, including in subtypes of MDS-EB2, MDS-EB1, and MDS-MLD, which implicates a poor prognosis in MDS.

In our study, univariable analysis showed the following items: age >65 years, bone marrow blasts ≥5%, with acquired CN-LOH, new aberrations detected by SNP-A, TGA value > the median (81.435 Mb), higher risk by IPSS-R-MC, higher risk by IPSS-R-SNP-A all had poorer prognosis. More critically, multivariable analysis showed that age >65 years and higher risk by IPSS-R-SNP-A were independent predictors of inferior OS in MDS patients. Tiu et al. also reported that new abnormalities detected by SNP-A predicted poor prognosis in MDS patients [7]. Yeung et al. showed that acquired CN-LOH and the median TGA values predicted poor prognosis in MDS patients [33]. The above results prove that SNP-A karyotyping can not only improve the detection yield of chromosomal aberrations, but also the prognostic stratification of MDS patients.

However, compared with MC, SNP-A also have some shortcomings. Firstly, it is impossible to detect balanced chromosomal variations such as translocations and inversions. Secondly, when there are complex karyotype abnormalities, SNP-A cannot distinguish the clone sources of various mutations, while MC can capture the specific balanced or unbalanced chromosomal mutations of each clone more accurately from the perspective of a single cell. The complementary effects of the two techniques have been confirmed in large sample size study of MDS patients [34].

For the last decade, several criteria of prognostic stratification for MDS have been developed including IPSS, WPSS, and IPSS-R [15, 35], which based on haematologic parameters and cytogenetic abnormalities. Somatic gene mutations had not yet used in the risk stratification of patients with MDS. In recent years, gene sequencing of patients with MDS is becoming increasingly accessible. The integration of clinical data with diagnostic genome profiling improves the accuracy of currently available prognostic scores [36, 37]. Bernard et al. [38] developed a new Molecular International Prognostic Scoring System for Myelodysplastic Syndromes(IPSS-M) based on haematologic, cytogenetic, and molecular genetic features. Using somatic mutations of 31 genes, a multivariable analysis identified TP53^multihit^, FLT3 mutations, and MLL^PTD^ as top genetic predictors of adverse outcomes, SF3B1 mutations were associated with favourable outcomes. They further derived 6 IPSS-M risk categories with prognostic differences. It needs be validated by further clinical studies. In the 5th edition of the World Health Organisation Classification of Haematolymphoid Tumours, -5q, SF3B1, TP53 are set as MDS molecular genetic grouping criteria [39]. It seems logical to perform molecular genetic test (either gene panel or NGS) for every MDS patient. However, pathogenic genomic alterations of MDS are multitype including sequence variations, segmental deletions, copy neutral loss of heterozygosity, and point mutations, the sequencing analysis often need to couple with a technique to detect copy number status, usually with comparative genomic hybridisation (aCGH) or SNP-A. In addition, NGS analyses need to be considered on its cost-effect. We believe that SNP-A for MDS is still significant methodology even in the NGS era. At present, SNP-A plus MDS related gene panel test may be an optimal choice.

In conclusion, our study illustrated that SNP-A karyotyping may complement metaphase cytogenetic findings and probably further improve the diagnostic yield and provide more precise prognostic stratification in MDS patients. However, SNP-A may not completely replace MC because of its inability to detect balanced translocation and to detect different clones. From a practical point of view, we recommend the concurrent use of SNP-A and MC in the initial karyotypic evaluation for MDS patients on diagnosis and prognosis stratification.

## Data Availability

The datasets used and/or analysed during the study are available from the corresponding author on reasonable request.
